# Endometriosis and Ovarian Cancer Risk: A Systematic Review of Epidemiological Studies

**DOI:** 10.3389/fsurg.2014.00014

**Published:** 2014-05-08

**Authors:** Menelaos Zafrakas, Grigorios Grimbizis, Anna Timologou, Basil C. Tarlatzis

**Affiliations:** ^1^1st Department of Obstetrics and Gynecology, Medical School, Aristotle University of Thessaloniki, Thessaloniki, Greece; ^2^School of Health and Medical Care, Alexander Technological Educational Institute of Thessaloniki, Thessaloniki, Greece

**Keywords:** endometriosis, ovarian cancer, causal association, ovarian tumor, ovarian neoplasia

## Abstract

**Background:** A possible etiological association between endometriosis and ovarian cancer has been repeatedly reported in the literature.

**Objective:** Our aim was to evaluate published epidemiological data on this issue.

**Review Methods:** We conducted an extensive search of the literature in MEDLINE, of articles ever published until February 2014, using the key-words “endometriosis” and “ovarian” and one of the following terms in the title: “cancer” or “malignancy” or “malignant” or “tumor” or “neoplasia” or “neoplasm” or “transformation.” Retrieved papers were checked for further relevant publications.

**Results:** Overall, our search yielded 1 prospective cohort study, 10 retrospective cohort, and 5 case–control studies. A meta-analysis of these studies was not considered to be appropriate, due to differences in data reporting, study design, and adjustment for confounding factors.

**Limitations:** The main limitation of studies found, with one exception, was the lack of operative confirmation of endometriosis.

**Conclusion:** An association of endometriosis with clear-cell and endometrioid ovarian cancer was a consistent finding in most studies. On the other hand, existing epidemiological evidence linking endometriosis with ovarian cancer is insufficient to change current clinical practice. Prospective cohort studies, with prior laparoscopic confirmation, localization, and staging of endometriosis are needed, in order to further clarify this issue.

## Introduction

Endometriosis, the presence and growth of endometrial-like glandular epithelium and stroma outside the uterus, is a common, chronic, estrogen-dependent, benign gynecologic disease (1). The prevalence of endometriosis is estimated to be between 10 and 15% among women of reproductive age, but may be as high as 20–30% in women with infertility, and 40–60% in women with chronic pelvic pain (2). Its true prevalence however is unknown, given that a laparotomy or laparoscopy is required for diagnosis (1, 3, 4). Endometriosis is uncommon before the onset of menses and it usually regresses after menopause. Nowadays, endometriotic lesions are identified mainly at laparoscopy, located in the ovaries and the pouch of Douglas (1, 4); rare extraperitoneal lesions have been also reported (5, 6).

Theories on the etiopathogenesis of endometriosis fall into five categories: the most accepted theory of retrograde menstruation (Sampson’s theory), celomic metaplasia, the origin from embryonic cell rests, the induction theory, and lymphatic and vascular dissemination (7–9). Though endometriosis is clearly a benign condition, it shares characteristics often encountered in malignancy, such as local and distant dissemination, cell-invasion, and damage of adjacent tissues (2, 4). Endometriosis, however, does not have catabolic consequences and certainly it is not lethal (8).

Sampson was the first to report a case of ovarian cancer arising in endometrial tissue in that organ (10). Since then, the hypothesis of a possible etiological association between endometriosis and ovarian cancer has been studied extensively. Endometriosis and ovarian cancer share several common predisposing factors, including early age at menarche, short intervals between menses, and late age of menopause and nulliparity. On the other hand, they are both inversely associated with tubal ligation, hysterectomy, use of oral contraceptives, and pregnancy (11). Interestingly, tubal ligation is associated with a lower risk of ovarian cancer, particularly clear-cell and endometrioid carcinoma (11–14). This association is in line with the reported correlation of endometriosis specifically with these two histological types of ovarian cancer in epidemiological studies (1) and the theory of retrograde menstruation for the etiopathogenesis of endometriosis (11).

Despite numerous studies published to date assessing the potential link between endometriosis and ovarian cancer, a firm causal association has not been established yet. Given its prevalence, such an association could lead to a reappraisal of the current management of endometriosis (1). The aim of the present study was to conduct a systematic review of published epidemiological studies and critically evaluate existing data, from an evidence-based perspective.

## Methods

An initial extensive computerized literature search for articles ever published until October 2011 was carried out in the bibliographic databases MEDLINE and EMBASE, using the key-words: “endometriosis” and “ovarian cancer” in the fields “title/abstract.” A complementary literature search for articles ever published until February 2014 was carried out in MEDLINE, using the key-words “endometriosis” and “ovarian” and one of the following words in the title: “cancer” or “malignancy” or “malignant” or “tumor” or “neoplasia” or “neoplasm” or “transformation.” Both search strategies were unlimited for language and year of publication.

The primary inclusion criteria were epidemiological studies reporting the incidence of ovarian cancer in women with a history of endometriosis, and epidemiological studies reporting the incidence of endometriosis in ovarian cancer patients. The following study characteristics were used as exclusion criteria of identified studies: molecular studies in the field of clinically orientated basic science, histopathological studies, case series, case reports, and reviews of the literature.

The titles of studies were screened first, and after exclusion of irrelevant publications, the abstract content was evaluated, followed by evaluation of the main text content. The lists of references from included studies, together with those from relevant review articles were hand-searched in order to screen for additional articles possibly meeting the inclusion criteria.

Studies meeting the inclusion criteria were initially evaluated in full text, and those meeting the exclusion criteria were excluded. Studies included in the final assessment were critically evaluated for possible bias according to study design, including selection, detection, recall, and/or publication bias.

## Results

### Literature search results

The initial extensive literature search for articles ever published until October 2011 in MEDLINE and EMBASE, using as search terms “endometriosis” and “ovarian cancer” in the fields “title/abstract” was conducted by one author (Anna Timologou), and yielded 1,049 studies. After screening the titles, 939 articles were excluded, and from the remaining 110, evaluation of the abstract content by two authors (Menelaos Zafrakas and Anna Timologou) led to exclusion of 77 further publications. It is noteworthy, that 30 of the excluded articles were case reports, and 17 were reviews of the literature. The formal published version of the remaining 33 studies, together with that of the 17 review articles was further evaluated by three authors (Anna Timologou, Menelaos Zafrakas, and Grigorios Grimbizis).

The complementary search for articles ever published until February 2014 in MEDLINE using the aforementioned multiple key-words in the title was conducted by one author (Menelaos Zafrakas), and yielded 152 articles. Among these articles, 127 were excluded, due to title and abstract content or if they had already been identified with the extensive search. Hence, 25 articles were further evaluated in full text.

Altogether, 58 articles – 33 identified by the initial extensive literature search plus 25 identified by the complementary search – were further evaluated in full text. Forty-two of these studies were excluded, according to the aforementioned exclusion criteria, i.e., molecular studies in the field of clinically orientated basic science, histopathological studies, case series, case reports, and reviews of the literature. Hence, the extensive along with the complementary literature search yielded 16 publications meeting the inclusion criteria: 5 case–control (15–19), 1 prospective cohort (21), and 11 publications on retrospective cohort studies (20, 22–31). The content of 1 out of 11 publications on retrospective cohorts was included in another (26, 27), leaving in essence 10 different retrospective cohort studies. A meta-analysis was not considered to be appropriate, due to differences between studies in reporting of data, study design, and adjustment for confounding factors.

### Case–control studies

Table 1 presents an overview of case–control studies, including study design, number of controls, endometriosis and ovarian cancer cases, and the odds ratio found in each case–control study (14–18).

**Table 1:** **Overview of case–control studies assessing the association between endometriosis and ovarian cancer**.

Publication	Study design	Number of endometriosis cases	Number of ovarian cancer cases	Number of controls	Association
Ness et al. (15)	Case–control	66 (CG 85)	767	1367	OR 1.7 (95% CI 1.2–2.4)
Ness et al. (16)	Case–control	51 (CG 39)	3,627	5,229	OR 1.73 (95% CI 1.10–2.71)
Borgfeldt and Andolf (17)	Case–control	28,163^a^	81	84,489	OR 1.34 (95% CI 1.03–1.75)
Modugno et al. (18)	Case–control	177 (CG 184)	2,098	2953	OR 1.32 (95% CI 1.06–1.65)
Pearce et al. (19)	Case–control	738 (CG 818)	7,911	13,226	OR 1.49 (95% CI 1.34–1.65)

CG, control group; OR, odds ratio;

^a^three controls for each case

Ness et al. (15), conducted, a population-based case–control study with 767 ovarian cancer patients (cases) and 1,367 controls, based on personal interviews of participants. After adjustment for various possible confounding factors, women with ovarian cancer had a higher likelihood of reporting a previous personal history of endometriosis (odds ratio – OR 1.7, 95% confidence interval – CI 1.2–2.4).

Later on, Ness and co-workers (16) analyzed pooled data from eight case–control studies on infertility, including 5,207 ovarian cancer cases and 7,705 controls, based on personal interviews or questionnaires. Again, after adjustment for possible confounding factors, endometriosis was associated with an increased risk of ovarian cancer (OR 1.73, 95% CI 1.10–2.71). Furthermore, endometriosis appeared to be associated with endometrioid/clear-cell ovarian tumors (OR 3.41, 95% CI 1.94–5.99).

Borgfeld and Andolf (17) identified 28,163 women discharged from hospital with diagnosis of endometriosis, by using the Swedish Hospital Discharge Register, and three controls were matched to each case. All incident cancers diagnosed among cases and controls were then matched, by using the National Swedish Cancer Register. A weak association between endometriosis and ovarian cancer was noted (OR 1.34, 95% CI 1.03–1.75).

By using a different methodological approach, Modugno and co-workers (18) analyzed pooled data on self-reported history of endometriosis from four population-based case–control studies of incident epithelial ovarian cancer, with a total number of 2,098 ovarian cancer cases and 2,953 control subjects. Multivariate unconditional logistic regression analysis showed again a weak association between endometriosis and ovarian cancer, with OR 1.32 (95% CI, 1.06–1.65).

The most recently published case–control study was conducted by Pearce and co-workers, for the Ovarian Cancer Association Consortium (19). Pooled data from 13 ovarian cancer case–control studies were analyzed, using logistic regression, in order to assess the association between self-reported endometriosis and ovarian cancer risk. A history of endometriosis was reported by 818 out of 13,226 controls, 738 out of 7,911 women with invasive ovarian cancer, and 168 out of 1,907 women with borderline ovarian tumors. Self-reported endometriosis was associated with significantly (*p* < 0.0001) increased risk of invasive ovarian cancer (OR 1.49, 95% CI 1.34–1.65), and the following subtypes: clear-cell (OR 3.05, 95% CI 2.43–3.84), low-grade serous (OR 2.11, 95% CI 1.39–3.20), and endometrioid invasive ovarian carcinoma (OR 2.04, 95% CI 1.67–2.48). On the other hand, there was no association between endometriosis and borderline tumors, as well as with mucinous, and high-grade serous invasive ovarian cancer.

### Cohort studies

In Table 2, an overview of cohort studies is presented, including study design, numbers of endometriosis and ovarian cancer cases, mean follow-up period, and the association found in each study in terms of OR, standard incidence ration (SIR), relative risk (RR), or Hazard Ratio (HR) (20–31).

**Table 2:** **Overview of cohort studies assessing the association between endometriosis and ovarian cancer**.

Publication	Study design	Number of endometriosis cases	Number of ovarian cancer cases	Mean follow-up (in years)	Association
Brinton et al. (20)	Retrospective	20,686	29	11.4	SIR 1.9 (95% CI 1.3–2.8)
Olson et al. (21)	Prospective	1,392	3	13	RR 0.8 (95% CI 0.2–1.4)
Brinton et al. (22)	Retrospective	1,919	13	18.8	SIR 2.48 (95% CI 1.3–4.2)
Brinton et al. (23)	Retrospective	50	2,491	<1, 1–4, >5	RR 1.69 (95% CI 1.27–2.25)
Melin et al. (24)	Retrospective	64,492	122	12.7	SIR 1.43 (95% CI 1.19–1.71)
Melin et al. (25)	Retrospective	63,630	134	13.4	SIR 1.37 (95% CI 1.14–1.62)
Kobayashi et al. (26, 27)	Pro/retrospective	6,398	46	12.8	SIR 8.95 (95% CI 4.12–15.3)
Gemmill et al. (28)	Retrospective	4,331	10	–	OR 3.43 (95% CI 1.74–6.54)
Aris (29)	Retrospective	2,521	41	9	RR^a^ 1.6 (95% CI 1.12–2.09)
Stewart et al. (30)	Retrospective	2,978	38	16.9	HR 2.33 (95% CI 1.02–5.35)
Buis et al. (31)	Retrospective	3,657	34	–	HR 12.4 (95% CI 2.8–54.2)

SIR, standard incidence ratio; RR, relative risk; OR, odds ratio;

*^a^RR, rate ratio; HR, hazard ratio*.

Brinton and co-workers (20) carried out a retrospective study by linking the records of 20,686 women hospitalized for endometriosis, between 1969 and 1983, registered in the nationwide Swedish Inpatient Register, with data from the National Swedish Cancer Registry through 1989. The mean follow-up period was 11.4 years. The risk of developing ovarian cancer was mildly increased (SIR 1.9, 95% CI 1.3–2.8), and it was particularly elevated with a long-standing history of ovarian endometriosis (SIR 4.2, 95% CI 2.0–7.7).

In a prospective cohort study by Olson et al. (21), the authors examined whether self-reported diagnosis of endometriosis was associated with increased risk of ovarian among various cancers. Among 37,434 participants, 1,392 (3.8%) reported a history of endometriosis. By using Cox proportional hazards regression models, and after 13 years of follow-up, no association was found between endometriosis and the risk of ovarian carcinoma (RR 0.8, 95% CI 0.2–2.4). However, the number of ovarian cancer cases in this study was rather low (*n* = 3).

In a retrospective observational cohort study, Brinton et al. (22) analyzed data of 12,193 women evaluated for infertility between 1965 and 1988, in five large reproductive endocrinology practices in the USA. A significantly higher rate of ovarian cancer than the general population was found in this cohort of infertility patients, and women with endometriosis had the highest risk (SIR 2.48, 95% CI 1.3–4.2). Compared with women with secondary infertility without endometriosis, patients with primary infertility and endometriosis had a RR of 2.72 (95% CI 1.1–6.7).

In another retrospective cohort study, Brinton et al. (23) assessed, among other hypotheses, the association between hospital and outpatient admissions for endometriosis and the development of ovarian cancer in Denmark, between 1978 and 1998, by using record linkage techniques. This was a population-based cohort with more than 99,000 women, including 2,491 women with ovarian cancer and 860 with borderline ovarian tumors. Five or more years after its diagnosis, endometriosis appeared to be associated with the development of ovarian cancer (RR 1.69, 95% CI 1.27–2.25); this association was restricted, however, to endometrioid (RR 2.53, 95% CI 1.19–5.38) and clear-cell (RR 3.37, 95% CI 1.24–9.14) ovarian cancers.

In a retrospective cohort study, expanding the previous study of Brinton et al. (20), Melin et al. (24) identified 64,492 women discharged from hospital, with diagnosis of endometriosis between 1969 and 2000, by using the National Swedish Inpatient Register. Data were then linked to the National Swedish Cancer Register, and 122 ovarian cancer cases were identified. An elevated risk for ovarian cancer was found (SIR 1.43, 95% CI 1.19–1.71); the risk was even higher among women with early-diagnosed (SIR 2.01, 95% CI 1.26–3.05) and long-standing endometriosis (SIR 2.23, 95% CI 1.36–3.44). Interestingly, women who had hysterectomy before or at the time of the endometriosis diagnosis did not have an increased ovarian cancer risk.

In another study in the same cohort, Melin et al. (25) used the same Swedish Registers, in a slightly longer period (1969–2002), and data were additionally linked to the Swedish Multi-Generation Register in order to adjust for parity and age at first birth. A total of 63,630 women with endometriosis were included in the study, and 134 ovarian cancer cases were identified. Endometriosis was associated with an elevated risk of ovarian cancer (SIR 1.37, 95% CI 1.14–1.62). There was a non-significant decrease in the risk of ovarian cancer with increasing parity for women with endometriosis.

In a cohort study with prospective and retrospective components, Kobayashi et al. (26, 27) assessed the risk of ovarian cancer development among 6,398 women with ultrasonographically diagnosed “ovarian endometriomas,” in Japan. The median follow-up period was 12.8 years, and 46 incident ovarian cancers were identified. The ovarian cancer risk was found to be elevated significantly among patients with ovarian endometrioma (SIR 8.95, 95% CI 4.12–15.3). The risk did not increase with increasing follow-up duration, whereas it increased with increasing age at endometrioma diagnosis (SIR 13.2, 95% CI 6.90–20.9, in women above 50 years of age) (26). Clear-cell carcinoma (39%) and endometrioid adenocarcinoma (35%) were commonly observed among women with ovarian cancer (27).

In a US study of self-reported survey data in 4,331 women reporting surgically diagnosed endometriosis, Gemmill et al. (28) identified 10 cases of ovarian cancer (0.2%). Ovarian cancer was significantly more common than in the general population (OR 3.43, 95% CI 1.74–6.54).

In a retrospective study, Aris (29) collected data of women diagnosed with endometriosis, ovarian cancer, or both, between 1997 and 2006, from a population of a region in Quebec, Canada. A total of 2,521 patients had endometriosis, 292 ovarian cancer, and 41 had both endometriosis and ovarian cancer. Women with endometriosis appeared to have an increased risk of ovarian cancer (Rate Ratio 1.6, 95% CI 1.12–2.09). Histopathological analyses showed the predominance of endometrioid (24.4%) and clear-cell (21.9%) types in endometriosis-associated ovarian cancer.

Recently, Stewart et al. (30) carried out a retrospective study, in a cohort of 21,646 infertility patients in Western Australia between 1982 and 2002. Data were extracted from the Hospital Morbidity Data System and the Reproductive Technology Register, and linked to the Midwives Notifications System, the Deaths Register, and the Western Australia Cancer Registry. A total of 2,978 women had endometriosis, and 38 ovarian cancer. The mean follow-up period was 16.9 years. Using multivariate analysis, women with endometriosis appeared to have an increased risk of ovarian cancer (HR 2.33, 95% CI 1.02–5.35); the risk was even higher in women who did not have a recorded birth (HR 3.11, 95% CI 1.13–8.57), whereas it was lower in women who gave birth (HR 1.52, 95% CI 0.34–6.75).

Finally, Buis et al. (31) conducted a nationwide, historic cohort study among women with subfertility problems, between 1980 and 1995 in the Netherlands, including 3,657 women with endometriosis and 5,247 without evidence of endometriosis. In contrast to previous studies, diagnosis was pathologically confirmed in 78% of endometriosis cases. The Dutch Pathology Database and the Netherlands Cancer Registry were linked to assess the occurrence of ovarian cancer and ovarian borderline tumors in these patients, between 1989 and 2007. Women with endometriosis appeared to have an increased risk of ovarian cancer (HR 12.4, 95% CI 2.8–54.2) and ovarian borderline tumors (HR 5.5, 95% CI 1.5–20.2). Interestingly, when information from the pathology database were excluded, a lower risk was found for both ovarian cancer (HR 4.3, 95% CI1.6–11.2) and borderline tumors (HR 1.9, 95% CI 0.6–5.8), suggesting that estimates from studies with self-reported endometriosis may be too low, due to possible misclassification bias. However, the main limitations of this study were the low numbers of ovarian cancer cases, leading to wide confidence intervals, and the inclusion of infertility patients only.

## Discussion

In this systematic review, we have identified five case–control studies (Table 1) (15–19), assessing the potential association between endometriosis and ovarian cancer. Because of this type of study design, all five studies have the inherent limitation of potential selection bias (32, 33). In the study of Borgfeldt and Andolf (17), an additional source of selection bias, as well as detection bias might have been the use of a hospital discharge register to identify cases of endometriosis, since only serious cases needing hospitalization might have been included, while milder cases could have been left out of consideration. Another source of selection and/or publication bias might have been the parallel evaluation of other factors for possible association with ovarian cancer in some studies (15–18), including inflammation (15), infertility and the use of fertility drugs (16), benign ovarian cysts (17), and use of oral contraceptives (18). Furthermore, the use of personal interviews and/or questionnaires for self-reporting of endometriosis (15, 16, 18, 19) might have well led to recall bias.

The second category of epidemiological studies evaluated in the present systematic review was cohort studies (Table 2) (20–31). Unexpectedly, given the relatively large number of relevant publications, there was only one prospective cohort study identified (21). Moreover, the number of ovarian cancer cases in this study was very low, i.e., three cases of ovarian cancer among 1,392 endometriosis patients, suggesting that current evidence linking endometriosis with subsequent ovarian cancer formation is rather weak. All other cohort studies identified were retrospective with the inherent limitations of possible selection and detection bias (34). Certainly, the three studies based on data from the Swedish Hospital Discharge Register (20, 24, 25) have a high statistical power, as they have included tens of thousands of endometriosis cases (1). On the other hand, as mentioned above, this methodological approach might be an additional source of selection and detection bias, by taking under consideration only serious cases of endometriosis.

The principle weakness of most studies, both case–control and cohort, is the lack of operative and histological confirmation of endometriosis, since accurate diagnosis can be made only after laparoscopy or laparotomy (1, 4). Only in one recent retrospective cohort study (31), a substantial proportion of endometriosis cases were pathologically confirmed (in 78%); however the number of ovarian cancer cases in this study was rather low and the study included infertility patients only. Other published studies that have included a substantial number of surgically confirmed endometriosis were also retrospective and without adequate patient follow-up (35, 36). Furthermore, different methods of identification of endometriosis cases were used in epidemiological studies, such as self-reporting, hospital discharge registers, or ultrasound detection, making comparisons between those studies difficult.

Despite the above weak points, a consistent finding was that endometriosis was associated with a slight increase of ovarian cancer risk, by a factor ranging between 1.3 and 1.9 in terms of OR, SIR, or RR in most studies (see Tables 1 and 2). One notable exception was the study by Kobayashi et al. (26, 27), in which the SIR was 8.95. This finding was criticized, since some of the ovarian cysts detected ultrasonographically and characterized as “endometriomas” might have well been misdiagnosed, already existing ovarian tumors (1). Other exceptions, were noted in the studies of Brinton et al. (22) (SIR = 2.48), Stewart et al. (30) (HR = 2.33), and Buis et al. (31) (HR = 12.4), probably due to inclusion of infertility patients only. Finally, another exception is the relatively high OR exceeding 3.4 in the study of Gemmil et al. (28) in patient-reported, physician-diagnosed comorbid conditions in women with endometriosis. Though this study has certain limitations, including evaluation of multiple variables and self-reporting, it has the strong point, that only surgically diagnosed endometriosis cases have been included. Taken together, these findings suggest that the risk of ovarian cancer in endometriosis patients has been either over-estimated in some or under-estimated in other studies. In any case, at least a weak association has been consistently found in all studies, except the aforementioned cohort with a very low number of ovarian cancer cases (21).

Another consistent finding in some of the epidemiological studies (16, 19, 23, 27, 29), as well as in previous histopathological studies (36, 37), was the correlation of endometriosis with clear-cell and endometrioid ovarian cancer. This association, together with findings that ovarian cancer is usually diagnosed in patients with endometriosis in earlier stages and in a younger age, suggests that endometriosis-associated ovarian cancer might represent a distinct clinical entity (1, 11).

From an evidence-based point of view, existing epidemiological evidence linking endometriosis with ovarian cancer remains rather weak, especially given the lack of data from prospective studies. The consistent findings in most studies of an increased risk of ovarian cancer in endometriosis patients and the association with two specific subtypes of ovarian cancer are not strong enough to establish a clear-cut causal association between these two clinical entities, since a casual association due to common predisposing factors and/or pathophysiological mechanisms cannot be completely ruled out (1, 11). Moreover, at present, these associations do not seem to have clinical implications, and a change of current clinical practices cannot be justified.

On the other hand, whereas some criteria needed to establish causal inference are insufficient (strength, biological gradient, biological plausibility, analogy, and coherence), yet other criteria are fulfilled, including consistency, temporality, specificity, and experimental evidence in animal model (38). Considering the clinical implications surrounding the hypothesis that endometriosis might be etiologically linked to ovarian cancer, prospective cohort studies (3), preferably with prior laparoscopic confirmation, localization, and staging of endometriosis are needed, in order to clarify this issue.

## Conflict of Interest Statement

The authors declare that the research was conducted in the absence of any commercial or financial relationships that could be construed as a potential conflict of interest.

## References

[1] Munksgaard PS Blaakaer J. The association between endometriosis and gynaecological cancers and breast cancer: a review of epidemiologic data. Gynecol Oncol (2011) 123:157–63. 10.1016/j.ygyno.2011.06.017 21742370

[2] Sayesneh A Tsivos D Crawford R. Endometriosis and ovarian cancer: a systematic review. ISRN Obstet Gynecol (2011):140310. 10.5402/2011/140310 21789283 PMC3140029

[3] Somigliana E Vigano P Parazzini F Stoppelli S Giambattista E Vercellini P. Association between endometriosis and cancer: a comprehensive review and a critical analysis of clinical and epidemiological evidence. Gynecol Oncol (2006) 101:331–41. 10.1016/j.ygyno.2005.11.033 16473398

[4] Nezhat F Datta S Hanson V Pejovic T Nezhat C Nezhat C. The relationship of endometriosis and ovarian malignancy: a review. Fertil Steril (2008) 90:1559–70. 10.1016/j.fertnstert.2008.08.007 18993168

[5] Pados G Tympanidis J Zafrakas M Athanatos D Bontis JN. Ultrasound and MR-imaging in preoperative evaluation of two rare cases of scar endometriosis. Cases J (2008) 1:97. 10.1186/1757-1626-1-97 18706122 PMC2533002

[6] Dragoumis K Mikos T Zafrakas M Assimakopoulos E Stamatopoulos P Bontis J. Endometriotic uterocutaneous fistula after cesarean section: a case report. Gynecol Obstet Invest (2004) 57:90–92. 10.1159/000075384 14671417

[7] Gazvani R Templeton A New considerations for the pathogenesis of endometriosis. Int J Gynaecol Obstet (2002) 76:117–26 10.1016/S0020-7292(01)00577-X 11818105

[8] Olive DL Schwartz LG Endometriosis. N Engl J Med (1993) 328:1759–69 10.1056/NEJM199306173282407 8110213

[9] Zafrakas M Tarlatzis BC Streichert T Pournaropoulos F Wölfle U Smeets SJ Genome-wide microarray gene expression, array-CGH analysis and telomerase activity in advanced ovarian endometriosis: high degree of differentiation rather than malignant potential. Int J Mol Med (2008) 21:335–44. 10.3892/ijmm.21.3.335 18288381

[10] Sampson JA Endometrial carcinoma of ovary arising in endometrial tissue in that organ. Arch Surg (1925) 10:1–72 10.1001/archsurg.1925.01120100007001

[11] Van Gorp T Amant F Neven P Vergote I Moerman P. Endometriosis and the development of malignant tumors of the pelvis. A review of literature. Best Pract Res Clin Obstet Gynaecol (2004) 18:349–71. 10.1016/j.bpobgyn.2003.03.001 15157647

[12] Rosenblatt KA Thomas DB. Reduced risk of ovarian cancer in women with a tubal ligation hysterectomy. The World Health Organization Collaborative Study of Neoplasia and Steroid Contraceptives. Cancer Epidemiol Biomarkers Prev (1996) 5:933–5. 8922304

[13] Miracle-McMahill HL Calle EE Kosinski AS Rodriguez C Wingo PA Thun MJ Tubal ligation and fatal ovarian cancer in a large prospective cohort study. Am J Epidemiol (1997) 145:349–57. 10.1093/oxfordjournals.aje.a009112 9054239

[14] Hankinson SE Hunter DJ Colditz GA Willett WC Stampfer MJ Rosner B Tubal ligation, hysterectomy, and risk of ovarian cancer. A prospective study. JAMA (1993) 270:2813–8. 10.1001/jama.270.23.2813 8133619

[15] Ness RB Grisso JA Cottreau C Klapper J Vergona R Wheeler JE Factors related to inflammation of the ovarian epithelium and risk of ovarian cancer. Epidemiology (2000) 11:111–7. 10.1097/00001648-200003000-00006 11021606

[16] Ness RB Cramer DW Goodman MT Kjaer SK Mallin K Mosgaard BJ Infertility, fertility drugs, and ovarian cancer: a pooled analysis of case-control studies. Am J Epidemiol (2002) 155:217–24. 10.1093/aje/155.3.217 11821246

[17] Borgfeldt C Andolf E. Cancer risk after hospital discharge diagnosis of benign ovarian cysts and endometriosis. Acta Obstet Gynecol Scand (2004) 83:395–400. 10.1111/j.0001-6349.2004.00305.x 15005789

[18] Modugno F Ness RB Allen GO Schildkraut JM Davis FG Goodman MT. Oral contraceptive use, reproductive history, and risk of epithelial ovarian cancer in women with and without endometriosis. Am J Obstet Gynecol (2004) 191:733–40. 10.1016/j.ajog.2004.03.035 15467532

[19] Pearce CL Templeman C Rossing MA Lee A Near AM Webb PM Association between endometriosis and risk of histological subtypes of ovarian cancer: a pooled analysis of case-control studies. Lancet Oncol (2012) 13:385–94. 10.1016/S1470-2045(11)70404-1 22361336 PMC3664011

[20] Brinton LA Gridley G Persson I Baron J Bergqvist A. Cancer risk after a hospital discharge diagnosis of endometriosis. Am J Obstet Gynecol (1997) 176:572–9. 10.1016/S0002-9378(97)70550-7 9077609

[21] Olson JE Cerhan JR Janney CA Anderson KE Vachon CM Sellers TA. Postmenopausal cancer risk after self-reported endometriosis diagnosis in the Iowa Women’s Health Study. Cancer (2002) 94:1612–8. 10.1002/cncr.10370 11920519

[22] Brinton LA Lamb EJ Moghissi KS Scoccia B Althuis MD Mabie JE Ovarian cancer risk associated with varying causes of infertility. Fertil Steril (2004) 82:405–14. 10.1016/j.fertnstert.2004.02.109 15302291

[23] Brinton LA Sakoda LC Sherman ME Frederiksen K Kjaer SK Graubard BI Relationship of benign gynecologic diseases to subsequent risk of ovarian and uterine tumors. Cancer Epidemiol Biomarkers Prev (2005) 14:2929–35. 10.1158/1055-9965.EPI-05-0394 16365012

[24] Melin A Sparén P Persson I Bergqvist A. Endometriosis and the risk of cancer with special emphasis on ovarian cancer. Hum Reprod (2006) 21:1237–42. 10.1093/humrep/dei462 16431901

[25] Melin A Sparén P Bergqvist A. The risk of cancer and the role of parity among women with endometriosis. Hum Reprod (2007) 22:3021–6. 10.1093/humrep/dem209 17855408

[26] Kobayashi H Sumimoto K Moniwa N Imai M Takakura K Kuromaki T Risk of developing ovarian cancer among women with ovarian endometrioma: a cohort study in Shizuoka, Japan. Int J Gynecol Cancer (2007) 17:37–43. 10.1111/j.1525-1438.2006.00754.x 17291229

[27] Kobayashi H Sumimoto K Kitanaka T Yamada Y Sado T Sakata M Ovarian endometrioma – risks factors of ovarian cancer development. Eur J Obstet Gynecol Reprod Biol (2008) 138:187–93. 10.1016/j.ejogrb.2007.06.017 18162283

[28] Gemmill JA Stratton P Cleary SD Ballweg ML Sinaii N. Cancers, infections, and endocrine diseases in women with endometriosis. Fertil Steril (2010) 94:1627–31. 10.1016/j.fertnstert.2009.07.1698 19945097 PMC2946463

[29] Aris A. Endometriosis-associated ovarian cancer: a ten year cohort study of women living in the Estrie Region of Quebec, Canada. J Ovarian Res (2010) 19:2. 10.1186/1757-2215-3-2 20205767 PMC2822768

[30] Stewart LM Holman CD Aboagye-Sarfo P Finn JC Preen DB Hart R. In vitro fertilization, endometriosis, nulliparity and ovarian cancer risk. Gynecol Oncol (2013) 128:260–4. 10.1016/j.ygyno.2012.10.023 23116937

[31] Buis CC van Leewen FE Mooij TM Burger CW OMEGA Project Group. Increased risk of ovarian cancer and borderline tumors in subfertile women with endometriosis. Hum Reprod (2013) 18:3358–69. 10.1093/humrep/det340 24014607

[32] Kopec JA Esdaile JM Bias in case-control studies. A review. J Epidemiol Community Health (1990) 44:179–86 10.1136/jech.44.3.179 2273353 PMC1060638

[33] Davies HT Crombie IK Bias in case-control studies. Hosp Med (2000) 61:279–81. 10858807 10.12968/hosp.2000.61.4.1875

[34] Davies HT Crombie IK Bias in cohort studies. Hosp Med (2000) 61:133–5. 10748794 10.12968/hosp.2000.61.2.1280

[35] Kontoravdis A Augoulea A Lambrinoudaki I Christodoulakos G Tzortziotis D Grammatikakis I Ovarian endometriosis associated with ovarian cancer and endometrial-endocervical polyps. J Obstet Gynaecol Res (2007) 33:294–8. 10.1111/j.1447-0756.2007.00527.x 17578358

[36] Takahashi K Kurioka H Irikoma M Ozaki T Kanasaki H Miyazaki K. Benign or malignant ovarian neoplasms and ovarian endometriomas. J Am Assoc Gynecol Laparosc (2001) 8:278–84. 10.1016/S1074-3804(05)60591-9 11342738

[37] Stern RC Dash R Bentley RC Snyder MJ Haney AF Robboy SJ. Malignancy in endometriosis: frequency and comparison of ovarian and extraovarian types. Int J Gynecol Pathol (2001) 20:133–9. 10.1097/00004347-200104000-00004 11293158

[38] Viganò P Somigliana E Parazzini F Vercellini P. Bias versus causality: interpreting recent evidence of association between endometriosis and ovarian cancer. Fertil Steril (2007) 88:588–93. 10.1016/j.fertnstert.2006.11.180 17320873

